# Development of loop-mediated isothermal amplification assay for rapid detection of genetically different wheat dwarf virus isolates

**DOI:** 10.1007/s11033-020-05846-0

**Published:** 2020-09-24

**Authors:** Katarzyna Trzmiel, Beata Hasiów-Jaroszewska

**Affiliations:** Department of Virology and Bacteriology, Institute of Plant Protection–National Research Institute (IPP-NRI), ul. Wł. Węgorka 20, 60-318 Poznań, Poland

**Keywords:** WDV-W, WDV-B, Wheat, Barley, Identification, LAMP technique

## Abstract

Wheat dwarf virus (WDV) is considered as one of the most common viruses on cereal crops. Recently, severe outbreaks of WDV have been observed especially on winter wheat in southwestern part of Poland. Moreover, the presence of genetically different WDV-barley-specific and WDV-wheat-specific forms (WDV-B and WDV-W, respectively) was confirmed. In this study, a loop-mediated isothermal amplification assay (LAMP) was developed for the first time for efficient and rapid detection of WDV-B and WDV-W in infected plants. The reaction was performed using a set of three primer pairs: WDVF3/WDVB3, WDVFIB/WDVBIP and WDVLoopF/WDVLoopB specific for coat protein coding sequence. The amplified products were analyzed by direct staining of DNA, gel electrophoresis and real-time monitoring of the amplification curves. The sensitivity of optimized reaction was tenfold higher in comparison with conventional PCR. LAMP assay developed here is a useful and practical method for the rapid detection of different WDV isolates and can be implemented by phytosanitary services.

## Introduction

*Wheat dwarf virus* (WDV) is a member of the genus *Mastrevirus* in the family *Geminiviridae*. The virus is transmitted by leafhoppers *Psammotettix alienus* (Dahlb.) [1] and *P. provincialis* [2] and it infects numerous species within the family *Poaceae* including important cereals (mainly wheat and barley) [3]. WDV induces yellowing of leaves, severe stunting and, in case of early infection, death of infected plants which may lead to yield losses up to 80% [4]. The virus was first reported in Czechoslovakia [5] and subsequently in many countries in Europe, Asia and North Africa [6]. Severe outbreaks of WDV have been reported in Sweden [7], Czech Republic [8], Austria [6] and recently also in Poland [9].

The virus has monopartite single-stranded circular (ss)DNA genome of ~2.7 kb which encodes four proteins: the movement protein (MP), the coat protein (CP) and two others associated with replication (Rep and RepA). Large and small intergenic regions (LIR and SIR, respectively) contain regulatory elements for viral replication and transcription [3]. The recently published results of the phylogenetic studies confirmed division of the global WDV population into two clearly separated WDV-wheat and WDV-barley-specific groups, according to their host. The studies have shown that the crucial regions for this division were MP and LIR [10]. Moreover, six WDV strains (A-E) have been distinguished based on sequence similarity between virus isolates and phylogenetic relationships among them [10].

To date, molecular detection of WDV is based on polymerase chain reaction (PCR), immunocapture (IC-PCR), real-time PCR, PCR and rolling circle amplification (RCA) methods combined with restriction fragment length polymorphism (RFLP) [11–16]. The promising alternative for above mentioned techniques is loop-mediated isothermal amplification of nucleic acids which has been developed for the rapid and efficient detection of many DNA and RNA viruses [17, 18]. LAMP assay amplifies a target sequence with high sensitivity and specificity under isothermal conditions (63–65 °C) and exhibits sensitivity similar to or even higher than the conventional PCR. Taking into account all the advantages of the LAMP assay, our objective was to develop and evaluate LAMP for the detection of WDV isolates collected from different hosts.

## Material and methods

The analyses were performed with DNA extracted from infected wheat, barley and triticale plants collected in 2012–2016 from different locations: Dłużec, Kobierzyce and Kondratowice in Dolny Śląsk as well as from Antoniny, Szelejewo and Strzelce in Wielkopolska region of Poland (Table 1). Total DNA was isolated using NucleoSpin® Plant II kit (Macherey–Nagel, Düren, Germany) according to manufacturer’s instructions. The DNA concentration and quality were estimated using a NanoDrop 2000 spectrophotometer (Nonodrop Technologies, Delaware, USA). In preliminary step, complete genome sequences of published, different WDV isolates: WDV-A (AJ783960), WDV-B (FJ620684), WDV-C (JQ647455), WDV-D (JN791096), WDV-E (AM040732) as well as two Polish isolates WDV-W (KM079154) and WDV-B (KM079155) were compared. Based on the result of multiple sequence alignments performed in ClustalW [19] CP region was chosen as a target for amplification in LAMP assay. The set of diagnostic primers (Table 2) were designed using LAMP Designer software (OptiGene, Horsham, UK). The specificity of LAMP primers was examined using 50 ng/µl of total DNA extracted from WDV-infected plants and from healthy wheat cv. Muszelka and barley cv. Bażant plants as the templates for reactions. LAMP assay was performed in total volume of 25 μl. The reaction mixture consisted of 15 μl Isothermal Mastermix ISO-001nd (Novazym, Poznan, Poland), 2 μl of 10 µM WDV FIP and WDV BIP, 1 µl of 10 µM WDV Loop-F and WDV Loop-B, 0.5 μl of 10 µM WDV F3 and WDV B3 primers, 1 µl of template DNA and 2 μl of sterile water. DNA samples from healthy plants and the reaction mix without a template were included as the negative controls. The tubes were incubated at 63 °C for 45 min in a thermoblock (Biometra, Göttingen, Germany). The LAMP products were analysed by electrophoretical separation in 1.5% agarose gel containing Midori Green DNA Stain (NIPPON Genetics Europe GmbH, Düren, Germany) as well as by direct visual inspection of color solution under UV light after the addition of 2 μl of SYBRGreen Dye (Thermo Fisher Scientific, Waltham, MA, USA). The reaction was also carried out using Isothermal Mastermix ISO-001 (Novazym) in a LightCycler 96 Instrument (Roche, Basel, Switzerland) and the results were estimated by analysis of amplification curves. The fluorescence data were obtained on the FAM channel (excitation at 470 nm, detection at 510 nm) in 30 min. The sensitivity of LAMP assay and conventional PCR were estimated and compared. For this purpose, 1 µl of tenfold serial dilutions (from 10^1^ to 10^–6^) of total DNA of WDV-Strz (adjusted to 50 ng/µl) were used as template for reactions. LAMP assay was performed in conditions described above and PCR was done with Dream Taq Green Master Mix (Thermo Fisher Scientific). The primer pair WDVuniw-F (CGCACTCGGCTTTTCGTGAGTG) and WDVuniw-R (CGCCAGGCGTAGTCGGAGG) were designed by Primer3software [20] based on conservative motifs of fragments of LIR and MP. The potential cross-reactivity of the oligonucleotides as well as their specificity were verified by basic local alignment search tool (BLAST) from website of the National Centre for Biotechnology Information (NCBI) (https://www.ncbi.nim.nih.gov). The PCR was performed in a final volume of 25 µl containing: 1 µl of DNA template with 12.5 µl of 2 × Dream Taq Master mix (Thermo Fisher Scientific), 1 µl of Primer Mix (10 µmol/µl each) and sterile Milli-Q water. Amplification was performed as follows: 94 °C for 2 min, 35 cycles of 94 ºC for 30 s, 55 °C for 30 s, 72 ºC for 30 s and a final cycle of 72 °C for 7 min. The detection limit of compared techniques was determined by electrophoresis in 1.5% agarose gel.

**Table 1:** Description of WDV isolates used in this study

Isolate name	Specification	Geographical origin	Host	Collection date	Accession no
WDV-Knd1	WDV-W	Kondratowice	Wheat	05/2013	KY781937
WDV-Kob	WDV-W	Kobierzyce	Wheat	06/2016	KY781939
WDV-Strz	WDV-W	Strzelce	Wheat	05/2013	MT460909
WDV-Ant	WDV-W	Antoniny	Wheat	04/2014	KY781933
WDV-Sz2	WDV-B	Szelejewo	Wheat	05/2012	KY781944
WDV-Dl	WDV-B	Dłużec	Barley	05/2016	KY781935
WDV-Sz4	WDV-W	Szelejewo	Triticale	05/2012	KY781946

**Table 2:** Primers used in LAMP techniques

Primer’s name	Primer sequence (5′-3′)
WDV F3	GTGAAGAGGAAGTGGTGC
WDV B3	CCACTGACACCACCTCTA
WDV FIP	CCGCTACGTAGTTGTAACGAGGGTGAACCTTGTCTCCGATG
WDV BIP	GCAAAGGTTTGCGGGTCACCCTTCTTAATGTCGCCTATCTT
WDV LoopF	CTTGGATCCGACCTTCTTCC
WDV LoopB	ACGGAGTGGATGAACACG

## Results and discussion

**Fig. 1:**
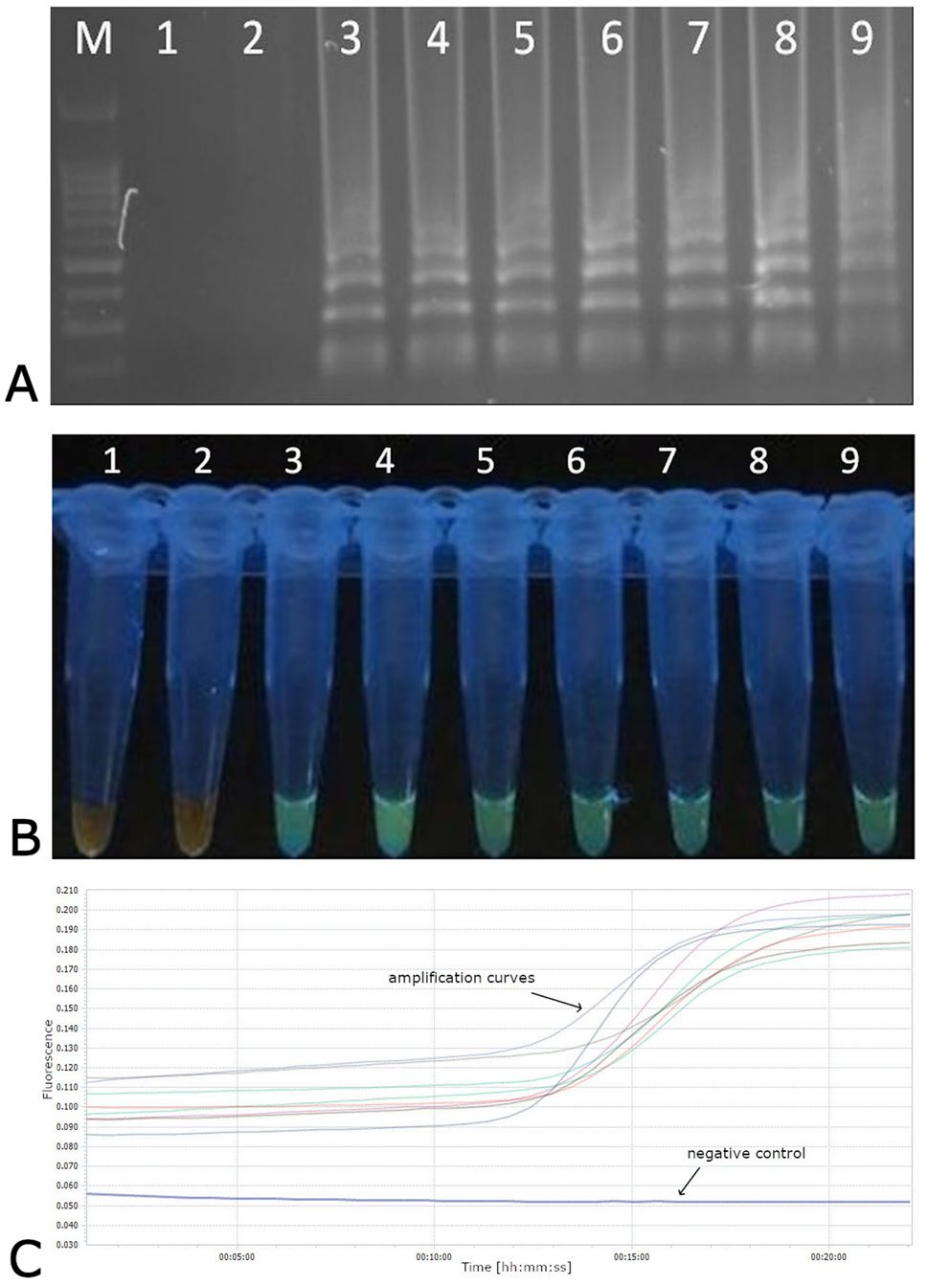
Detection of WDV isolates by LAMP. **a**, Analysis of LAMP products on agarose gels; lane M–100-bp DNA ladder (Novazym, Poznań, Poland), lane 1-negative control- sap of healthy barley, lane 2–sap of healthy wheat; lane 3- 9- WDV isolates **b**, Visual detection of LAMP products using SYBRGreen Dye (Thermo Fisher Scientific). Numbers correspond to the gel lanes in panel **a**. **c**) Real-time monitoring of LAMP assay of the WDV isolates in LightCycler 96 (Roche)

**Fig. 2:**
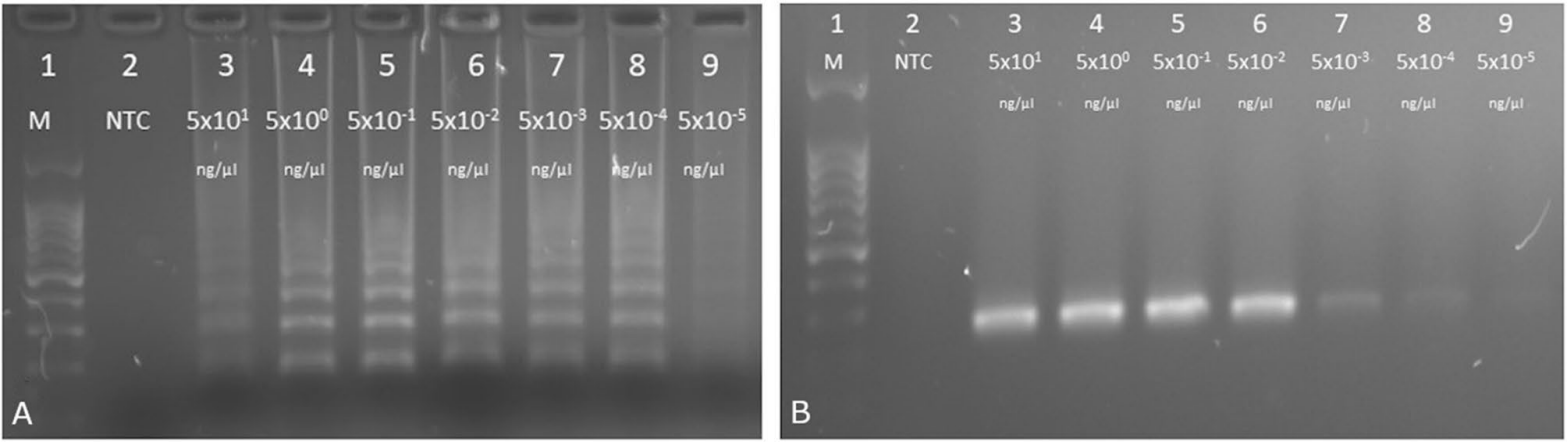
A comparison of sensitivity of LAMP and PCR methods for WDV detection. Detection limit of LAMP assay (**a**) and PCR technique (**b**). Lanes: 1–100-bp DNA ladder (Novazym); 2–NTC negative control (water); 3–9– correspond to serial tenfold dilutions of total DNA

WDV population can be clearly divided into barley and wheat forms [3, 6, 10, 14]. The genomes of these two forms share 84% nucleotide sequence identity however the *CP* gene is one of the most stable coding regions [21]. The comparative analysis of this region revealed ca. 83% nucleotide sequence identity between different barley- and wheat- infecting WDV isolates whereas up to 98% within the particular group [14, 21]. Taking into account the genetic diversity of the WDV population, the primers for the LAMP assay were designed based on the alignment of CP sequences. LAMP technique developed here was capable of the detection of both WDV-B and WDV-W forms isolated from different hosts in less than hour. In contrast to previously published differentiating methods [11–15] designed set of primers allow for the detection of genetically diverse WDV isolates in single reaction. The amplified products visible as ladder-like DNA fragments were obtained only for tested WDV samples whereas no amplicons were produced for the samples of healthy barley and wheat plants or for the water controls (Fig. 1a). Green color was observed in UV light after adding the dye only to WDV infected samples and no color changes were seen for the negative controls (Fig. 1b). Monitoring of LAMP reaction in real-time revealed rapid and specific detection of WDV in tested samples. The amplification plots was already observed between 10 and 20 min and only for samples with DNA of WDV (Fig. 1c). The specific PCR product (235 bp in size) was detectable up to 5 pg/µl of DNA while LAMP amplicons could be seen even for 500 fg/µl of total DNA indicating that LAMP assay was 10 times more sensitive compared to the conventional PCR (Fig. 2).

Obtained results and sensitivity of virus detection correspond with those presented by Kuan et al. [22] for *Squash leaf curl virus* (SLCV) belonging to *Begomovirus* genus in *Geminiviridae* family or with that reported by Kill et al. [18] for three virus species of *Curtovirus* genus also classified in *Geminiviridae* family. In conclusion, the results confirmed that LAMP assay is a high specific and suitable technique, faster and more sensitive than standard PCR. Moreover, it does not require specialized equipment and the reaction can be carried out with a simple heater or in water bath. All the features mentioned above confirm that optimised in this study LAMP assay is useful diagnostic tool for rapid identification of WDV infection in cereals. Furthermore, according to our knowledge it is the first report presenting the use of the LAMP assay for WDV detection.

## Data Availability

The data of this study are available from the corresponding author upon request.
